# Interpretation of coefficients in segmented regression for interrupted time series analyses

**DOI:** 10.1186/s12874-025-02556-8

**Published:** 2025-04-16

**Authors:** Yongzhe Wang, Narissa J. Nonzee, Haonan Zhang, Kimlin T. Ashing, Gaole Song, Catherine M. Crespi

**Affiliations:** 1 Department of Surgery, City of Hope Comprehensive Cancer Center, Duarte, CA 91010 USA; 2 Department of Population Sciences, City of Hope Comprehensive Cancer Center, Duarte, CA 91010 USA; 3 Department of Information Systems and Operations Management, Foster School of Business, University of Washington, Seattle, WA 98105 USA; 4 Department of Biostatistics, Fielding School of Public Health, University of California, Los Angeles, Los Angeles, CA 90095 USA

**Keywords:** Observational study, Interrupted time series design, Segmented regression, Healthcare policy evaluation, Coefficient interpretation

## Abstract

**Background:**

Segmented regression, a common model for interrupted time series (ITS) analysis, primarily utilizes two equation parametrizations. Interpretations of coefficients vary between the two segmented regression parametrizations, leading to occasional user misinterpretations.

**Methods:**

To illustrate differences in coefficient interpretation between two common parametrizations of segmented regression in ITS analysis, we derived analytical results and present an illustration evaluating the impact of a smoking regulation policy in Italy using a publicly accessible dataset. Estimated coefficients and their standard errors were obtained using two commonly used parametrizations for segmented regression with continuous outcomes. We clarified coefficient interpretations and intervention effect calculations.

**Results:**

Our investigation revealed that both parametrizations represent the same model. However, due to differences in parametrization, the immediate effect of the intervention is estimated differently under the two approaches. The key difference lies in the interpretation of the coefficient related to the binary indicator for intervention implementation, impacting the calculation of the immediate effect.

**Conclusions:**

Two common parametrizations of segmented regression represent the same model but have different interpretations of a key coefficient. Researchers employing either parametrization should exercise caution when interpreting coefficients and calculating intervention effects.

**Supplementary Information:**

The online version contains supplementary material available at 10.1186/s12874-025-02556-8.

## Background

The interrupted time series (ITS) design is an increasingly popular quasi-experimental design that is used to estimate the effectiveness of an intervention when a randomized trial is not feasible [1–7]. In an ITS design, observations are collected in a time series over a study period that includes intervals both before and after the introduction of an intervention, and these observations are contrasted to estimate the intervention’s effectiveness. ITS designs have been used widely in health services research, for example, in the evaluation of health policies and health care quality improvement interventions in real-world settings [2, 8–14].

The most widely used method of analyzing data from an ITS design study is segmented regression [1, 2, 4–6, 15, 16]. Segmented regression, also known as piecewise regression or broken-stick regression, is a method in regression analysis in which a series of observations is partitioned into intervals and a separate line segment is fit to each interval. The theoretical framework for estimating segmented regression dates back to the work of Quandt [17, 18]. The use of segmented regression for ITS dates back to its application in evaluating cross-sectional time series experiments in psychology [19].

There are two common parametrizations for segmented regression applied to ITS analyses, that of Bernal et al. [6, 7] and that of Wagner et al. [4]. Superficially, these two parametrizations appear similar, but they have important differences that impact the estimation of intervention effects, raising concerns about the potential for misinterpretation of results [20]. This paper investigates the two different parametrizations and their interpretations and illustrates the differences in interpretation by applying them to a real data set [7].

## Methods

### Parametrizations of segmented regression

To explain the two common parametrizations of segmented regression for ITS, we consider the setting of a single interrupted time series collected from one unit (for example, a single clinic) with a continuous outcome variable [3, 7]. The key features of the model equation are a variable for continuous time, a binary indicator denoting the presence of an intervention, and an outcome measure [1–3, 6, 7, 14, 15, 21]. Let $T$ represent continuous time measuring the duration since the study’s initiation, starting from 0, and let $\delta$ denote the time at which the intervention is introduced. ${X}_{t}$ represents a binary indicator denoting the presence or absence of an intervention at time $t$, equal to 0 for $T<\delta$ and 1 for $T\ge \delta$. Let ${y}_{t}$ denote the continuous outcome as measured at time $t$.

Bernal’s parametrization involves regressing the outcome ${y}_{t}$ on $T$, ${X}_{t}$, and their interaction [6, 7, 19, 22–25]. Bernal’s parametrization [7] is:

1 $${y}_{t}={\beta }_{0}+{\beta }_{1}T+{\beta }_{2}^{B}{X}_{t}+{\beta }_{3}{X}_{t}T$$

2 $$=\left\{\begin{array}{c}{\beta }_{0}+{\beta }_{1}T, T<\delta \\ {(\beta }_{0}+{\beta }_{2}^{B})+{(\beta }_{1}+{\beta }_{3})T, T\ge \delta \end{array}\right.$$

In this parametrization, ${\beta }_{0}$ is the intercept in the pre-intervention interval and represents the mean outcome level at the inception of the study ($T=0$). ${\beta }_{1}$ is the slope during the pre-intervention interval and represents the mean change in the outcome for a one unit increase in time. For the post-intervention interval, ${\beta }_{0}+{\beta }_{2}^{B}$ is the intercept and ${\beta }_{1}+{\beta }_{3}$ is the slope. Note that ${\beta }_{0}+{\beta }_{2}^{B}$ represents the outcome level at time 0 if we extrapolated the post-intervention regression line backwards in time. The coefficients ${\beta }_{2}^{B}$ and ${\beta }_{3}$ represent the differences in intercept and slope between the pre- and post-intervention intervals. Thus, this model allows for different linear regression models (different intercepts and different slopes) during the pre- and post-intervention intervals.

Two different aspects of an intervention effect can be captured with this segmented regression model [4, 5, 21, 26, 27]. One aspect is a change in the mean level of the outcome at time $\delta$, corresponding to an *immediate effect* of the intervention on the outcome. The other aspect is the change in slopes from pre- to post-intervention, which represents a longer-term, *gradual effect* of the intervention on the outcome. In Bernal’s parametrization, the gradual effect corresponds to the change in slopes, which is ${\beta }_{3}$ in Eq. (1). However, the immediate effect does not correspond to the difference in intercepts (${\beta }_{2}^{B})$ [4, 20]. Rather, the immediate effect is the difference in means between the pre- and post-intervention models at the start of the intervention at time $\delta$, which can be formulated as:

$$\begin{array}{ll}Change\;in\;Levels&={(\beta}_0+\beta_2^B)+{(\beta}_1+\beta_3)\delta-(\beta_0+\beta_1\delta)\\&=\beta_2^B+\beta_3\delta\end{array}$$

Hence in Bernal’s parametrization, ${\beta }_{2}^{B}$ is the difference in intercepts between the pre- and post-intervention models, that is, the vertical difference between the two regression lines at time 0, and the immediate effect is given by ${\beta }_{2}^{B}+{\beta }_{3}\delta$.

The parametrization of segmented regression advanced by Wagner is the same as Bernal’s parametrization except for the interaction term [4]. In Wagner’s parametrization, the interaction is the product of the binary intervention indicator and the time elapsed since the intervention’s implementation, $T-\delta$. The model is:

3 $${y}_{t}={\beta }_{0}+{\beta }_{1}T+{\beta }_{2}^{W}{X}_{t}+{\beta }_{3}{X}_{t}(T-\delta )$$

4 $$=\left\{\begin{array}{c}{\beta }_{0}+{\beta }_{1}T, T<\delta \\ ({\beta }_{0}+{\beta }_{2}^{W}-{\beta }_{3}\delta )+({\beta }_{1}+{\beta }_{3})T, T\ge \delta \end{array}\right.$$

Under this parametrization, the intercept and slope of the pre-intervention model are the same as for Bernal, but the intercept and slope of the post-intervention model are ${\beta }_{0}+{\beta }_{2}^{W}-{\beta }_{3}\delta$ and ${\beta }_{1}+{\beta }_{3}$, respectively. Thus, the two parametrizations differ in the parametrization of the intercept of the post-intervention model. The difference in intercepts between the pre- and post-intervention models is ${\beta }_{2}^{W}-{\beta }_{3}\delta$. For intervention effects, ${\beta }_{3}$ represents the gradual effect, as it does in Bernal’s parametrization. However, the immediate effect, quantified as the mean change in levels at time $\delta$, is given by:

$$\begin{array}{ll}Change\;in\;Levels& =\left({\beta }_{0}+{\beta }_{2}^{W}-{\beta }_{3}\delta \right)+\left({\beta }_{1}+{\beta }_{3}\right)\delta -\left({\beta }_{0}+{\beta }_{1}\delta \right)\\ & ={\beta }_{2}^{W}\end{array}$$

Consequently, in this parametrization, ${\beta }_{2}^{W}$ captures the difference in means at the start of the intervention’s implementation. Thus when researchers use Wagner’s parametrization, the immediate effect can be directly extracted from ${\beta }_{2}^{W}$.

It is important to highlight that the intercept and slope coefficients for the pre-intervention models in both parametrizations are the same. Additionally, the post-intervention slopes are the same, being represented by ${\beta }_{1}+{\beta }_{3}$ in both Eqs. (2) and (4). The intercept terms of the two parametrizations are different: ${\beta }_{0}+{\beta }_{2}^{B}$ in Eq. (2) and ${\beta }_{0}+{\beta }_{2}^{W}-{\beta }_{3}\delta$ in equation $\left(4\right).$ Assuming the post-intervention intercepts under the two parametrizations are equivalent, we can find that:

$$\begin{array}{l}{\beta }_{0}+{\beta }_{2}^{B}={\beta }_{0}+{\beta }_{2}^{W}-{\beta }_{3}\delta \\ {\beta }_{2}^{W}={\beta }_{2}^{B}+{\beta }_{3}\delta \end{array}$$

Hence, despite the differences between the two parametrizations, they should give the same estimate of the immediate effect of the intervention. In the next section, we show the alignment between the two parametrizations through the analytical expressions of the estimated coefficients. We summarize the interpretation of coefficients and intervention effects under the two different parametrizations in Table 1.

**Table 1:** Summary of interpretation of coefficients and intervention effects in segmented regression for interrupted time series analysis using parametrizations of Bernal et al. and Wagner et al

	**Bernal’s parametrization**	**Wagner’s parametrization**
Model equation	${y}_{t}={\beta }_{0}+{\beta }_{1}T+{\beta }_{2}^{B}{X}_{t}+{\beta }_{3}{X}_{t}T$	${y}_{t}={\beta }_{0}+{\beta }_{1}T+{\beta }_{2}^{W}{X}_{t}+{\beta }_{3}{X}_{t}(T-\delta )$
**Interpretations**	**Coefficients**	
Baseline level	${\beta }_{0}$	
Pre-intervention trend	${\beta }_{1}$	
Difference in intercepts	${\beta }_{2}^{B}$	${\beta }_{2}^{W}-{\beta }_{3}\delta$
Immediate effect (change in levels at intervention onset)	${\beta }_{2}^{B}+{\beta }_{3}\delta$	${\beta }_{2}^{W}$
Gradual effect (change in slopes after intervention)	${\beta }_{3}$	
Post-intervention trend	${\beta }_{1}+{\beta }_{3}$	

### Estimated coefficients

As observed, the parametrizations of segmented regression proposed by Wagner et al. and Bernal et al. have different model equations but correspond to the same pre- and post-intervention models. The two parametrizations also lead to different design matrices. The design matrix for Bernal’s parametrization is

$${\mathbf{X}}_{B}=\left[\begin{array}{cccc}1& {t}_{1}& 0& 0\\ 1& {t}_{2}& 0& 0\\ 1& {t}_{3}& 0& 0\\ \vdots & \vdots & \vdots & \vdots \\ 1& {t}_{m}& 0& 0\\ 1& {t}_{m+1}& 1& {t}_{m+1}\\ 1& {t}_{m+2}& 1& {t}_{m+2}\\ \vdots & \vdots & \vdots & \vdots \\ 1& {t}_{m+n}& 1& {t}_{m+n}\end{array}\right]$$

where the upper part of the matrix represents the pre-intervention period, and the lower part represents the post-intervention period. We assume that there are $m$ and $n$ observations in the pre- and post-intervention periods, respectively, for a total of $N=m+n$ observations. The design matrix for Wagner’s parametrization is

$${\mathbf{X}}_{W}=\left[\begin{array}{cccc}1& {t}_{1}& 0& 0\\ 1& {t}_{2}& 0& 0\\ 1& {t}_{3}& 0& 0\\ \vdots & \vdots & \vdots & \vdots \\ 1& {t}_{m}& 0& 0\\ 1& {t}_{m+1}& 1& {t}_{m+1}-\delta \\ 1& {t}_{m+2}& 1& {t}_{m+2}-\delta \\ \vdots & \vdots & \vdots & \vdots \\ 1& {t}_{m+n}& 1& {t}_{m+n}-\delta \end{array}\right]$$

Using design matrices ${\mathbf{X}}_{B}$ or ${\mathbf{X}}_{W}$, we can obtain the ordinary least squares estimates of regression coefficients ${\varvec{\beta}}=\left[{\beta }_{0}, {\beta }_{1}, {\beta }_{2}, {\beta }_{3}\right]{\prime}$ by solving the normal equations, obtaining $\widehat{{\varvec{\beta}}}={(\mathbf{X}}^{{\varvec{T}}}\mathbf{X}{)}^{-1}{\mathbf{X}}^{{\varvec{T}}}\mathbf{y}$ where $\mathbf{y} = [{y}_{1}, {y}_{2},\cdots ,{y}_{m},{y}_{m+1},\cdots ,{y}_{m+n}]{\prime}$ is the vector of the outcome variable. The covariance matrix for $\widehat{{\varvec{\beta}}}$ can be obtained as $\widehat{{\varvec{\Sigma}}}={\widehat{\sigma }}^{2}{(\mathbf{X}}^{{\varvec{T}}}\mathbf{X}{)}^{-1}$ where ${\widehat{\sigma }}^{2}$ represents the estimated residual, calculated as ${\widehat{\sigma }}^{2}=\frac{1}{N-p}(\mathbf{y}-{\mathbf{X}\widehat{{\varvec{\beta}}})}^{T}(\mathbf{y}-\mathbf{X}\widehat{{\varvec{\beta}}})$ where $p$ indicates the number of columns in the design matrix. We will show the estimates of ${\varvec{\beta}}$ and ${\varvec{\Sigma}}$ in ordinary algebra rather than matrix algebra.

$${{\varvec{\beta}}}_{0}\;{{\varvec{\beta}}}_{1}$$, and $${{\varvec{\beta}}}_{3}$$  

The estimates of ${\beta }_{0}$*,* ${\beta }_{1}$, and ${\beta }_{3}$ take the forms

$$\begin{array}{ll}{\widehat{\beta }}_{0}& =\frac{(\sum_{j=1}^{m}{t}_{j}{y}_{j})(\sum_{j=1}^{m}{t}_{j})-(\sum_{j=1}^{m}{y}_{j})(\sum_{j=1}^{m}{t}_{j}^{2})}{(\sum_{j=1}^{m}{t}_{j}{)}^{2}-m(\sum_{j=1}^{m}{t}_{j}^{2})}, \\ {\widehat{\beta }}_{1}& =\frac{(\sum_{j=1}^{m}{t}_{j})(\sum_{j=1}^{m}{y}_{j})-m(\sum_{j=1}^{m}{t}_{j}{y}_{j})}{(\sum_{j=1}^{m}{t}_{j}{)}^{2}-m(\sum_{j=1}^{m}{t}_{j}^{2})},\\ {\widehat{\beta }}_{3}& =\frac{(\sum_{j=m+1}^{m+n}{t}_{j})(\sum_{j=m+1}^{m+n}{y}_{j})-n(\sum_{j=m+1}^{m+n}{t}_{j}{y}_{j})}{(\sum_{j=m+1}^{m+n}{t}_{j}{)}^{2}-n(\sum_{j=m+1}^{m+n}{t}_{j}^{2})}-{\widehat{\beta }}_{1}, \\ & ={\widehat{\beta }}_{3,post}-{\widehat{\beta }}_{1},\end{array}$$

where ${\widehat{\beta }}_{3,post}$ represents the post-intervention slope such that ${\widehat{\beta }}_{3,post}= {\widehat{\beta }}_{1}+{\widehat{\beta }}_{3}$. The summations $j=1$ to $m$ and $j=m+1$ to $m+n$ represent the summation over observations from the pre- and post-intervention periods, respectively. Under both parametrizations, ${\widehat{\beta }}_{0}$ represents the mean outcome at study initiation and serves as the intercept in the pre-intervention model, ${\widehat{\beta }}_{1}$ represents the pre-intervention slope, and ${\widehat{\beta }}_{3}$ represents the difference in slopes between the pre- and post-intervention models. Note that ${\widehat{\beta }}_{0}$ and ${\widehat{\beta }}_{1}$ use only information from the pre-intervention period while ${\widehat{\beta }}_{3}$ uses observations from each period to estimate a period-specific slope and then takes the difference. The estimated variances of these coefficients are

$$\begin{array}{l}{var(\widehat{\beta }}_{0})={\widehat{\sigma }}^{2}\frac{\sum_{j=1}^{m}{t}_{j}^{2}}{m\sum_{j=1}^{m}({t}_{j}-{\overline{t} }_{m}{)}^{2}},\\ {var(\widehat{\beta }}_{1})={\widehat{\sigma }}^{2}\frac{1}{\sum_{j=1}^{m}({t}_{j}-{\overline{t} }_{m}{)}^{2}}, \\ {var(\widehat{\beta }}_{3})={\widehat{\sigma }}^{2}\left[\frac{1}{\sum_{j=1}^{m}({t}_{j}-{\overline{t} }_{m}{)}^{2}}+\frac{1}{\sum_{j=m+1}^{m+n}({t}_{j}-{\overline{t} }_{n}{)}^{2}}\right],\end{array}$$

where $${\overline{t} }_{m}=\frac{1}{m}\sum_{j=1}^{m}{t}_{j}$$ and $${\overline{t} }_{n}=\frac{1}{n}\sum_{j=m+1}^{m+n}{t}_{j}$$.

$${{\varvec{\beta}}}_{2}$$

The estimates of ${\beta }_{2}$ values for the two different parametrizations are:

$$\begin{array}{l}{\widehat{\beta }}_{2}^{B}=\frac{(\sum_{j=m+1}^{m+n}{t}_{j}{y}_{j})(\sum_{j=m+1}^{m+n}{t}_{j})-(\sum_{j=m+1}^{m+n}{y}_{j})(\sum_{j=m+1}^{m+n}{t}_{j}^{2})}{(\sum_{j=m+1}^{m+n}{t}_{j}{)}^{2}-n(\sum_{j=m+1}^{m+n}{t}_{j}^{2})}-{\widehat{\beta }}_{0}={\widehat{\beta }}_{2,post}^{B}-{\widehat{\beta }}_{0}, \\ {\widehat{\beta }}_{2}^{W}={\widehat{\beta }}_{2}^{B}+{\delta \widehat{\beta }}_{3}=\left({\widehat{\beta }}_{2,post}^{B}+\delta {\widehat{\beta }}_{3,post}\right)-\left({\widehat{\beta }}_{0}+\delta {\widehat{\beta }}_{1}\right),\end{array}$$where ${\widehat{\beta }}_{2,post}^{B}$ represents the post-intervention intercept under Bernal’s parametrization such that ${\widehat{\beta }}_{2,post}^{B}={\widehat{\beta }}_{0}+{\widehat{\beta }}_{2}^{B}$. ${\widehat{\beta }}_{2}^{B}$ corresponds to the difference in intercepts between the pre- and post-intervention models. On the other hand, ${\widehat{\beta }}_{2}^{W}$ corresponds to the difference in the mean outcome at the time of intervention implementation. The estimated variances for ${\widehat{\beta }}_{2}$ for the two parametrizations are

$$\begin{array}{l}{var(\widehat{\beta }}_{2}^{B})={\widehat{\sigma }}^{2}\left[\frac{\sum_{j=1}^{m}{t}_{j}^{2}}{m\sum_{j=1}^{m}({t}_{j}-{\overline{t} }_{m}{)}^{2}}+\frac{\sum_{j=m+1}^{m+n}{t}_{j}^{2}}{n\sum_{j=m+1}^{m+n}({t}_{j}-{\overline{t} }_{n}{)}^{2}}\right],\\ {var(\widehat{\beta }}_{2}^{W})={\widehat{\sigma }}^{2}\left[\frac{\sum_{j=1}^{m}({t}_{j}-\delta {)}^{2}}{m\sum_{j=1}^{m}({t}_{j}-{\overline{t} }_{m}{)}^{2}}+\frac{\sum_{j=m+1}^{m+n}({t}_{j}-\delta {)}^{2}}{n\sum_{j=m+1}^{m+n}({t}_{j}-{\overline{t} }_{n}{)}^{2}}\right].\end{array}$$

Standard errors are obtained as the square root of the variances. For estimates of linear combinations of coefficients, such as ${\beta }_{2}^{B}+{\beta }_{3}\delta$ and ${\beta }_{2}^{W}-{\beta }_{3}\delta$, the covariance between ${\beta }_{2}$ and ${\beta }_{3}$ is also needed to obtain the standard error. We omit this formula. All standard errors can be calculated in standard software.

## Results

### Illustration

We illustrate the differences in the two parametrizations using a dataset provided by Barone-Adesi et al. [28] and analyzed by Bernal et al. [7]. The objective of Bernal et al.’s study was to assess the effectiveness of a policy that banned smoking in all indoor public places in Sicily, Italy. The policy implementation began in January 2005. The researchers adopted an ITS design and collected data between 2002 and 2006 on the standardized rates of acute coronary episodes (ACE) in Sicily per month. The standardized ACE rates were computed by dividing the monthly frequency of ACE hospital admissions in Sicily by the age-standardized population per person-year. We expressed the outcome as standardized ACE rates per 1000. There were 36 and 22 observations of standardized ACE rates in the pre- and post-intervention periods, respectively. Our focus is on illustrating the two parametrizations rather than providing a detailed analysis of these data, as was done by Bernal et al. [7]. Hence, we do not present a complete analysis.

**Fig. 1:**
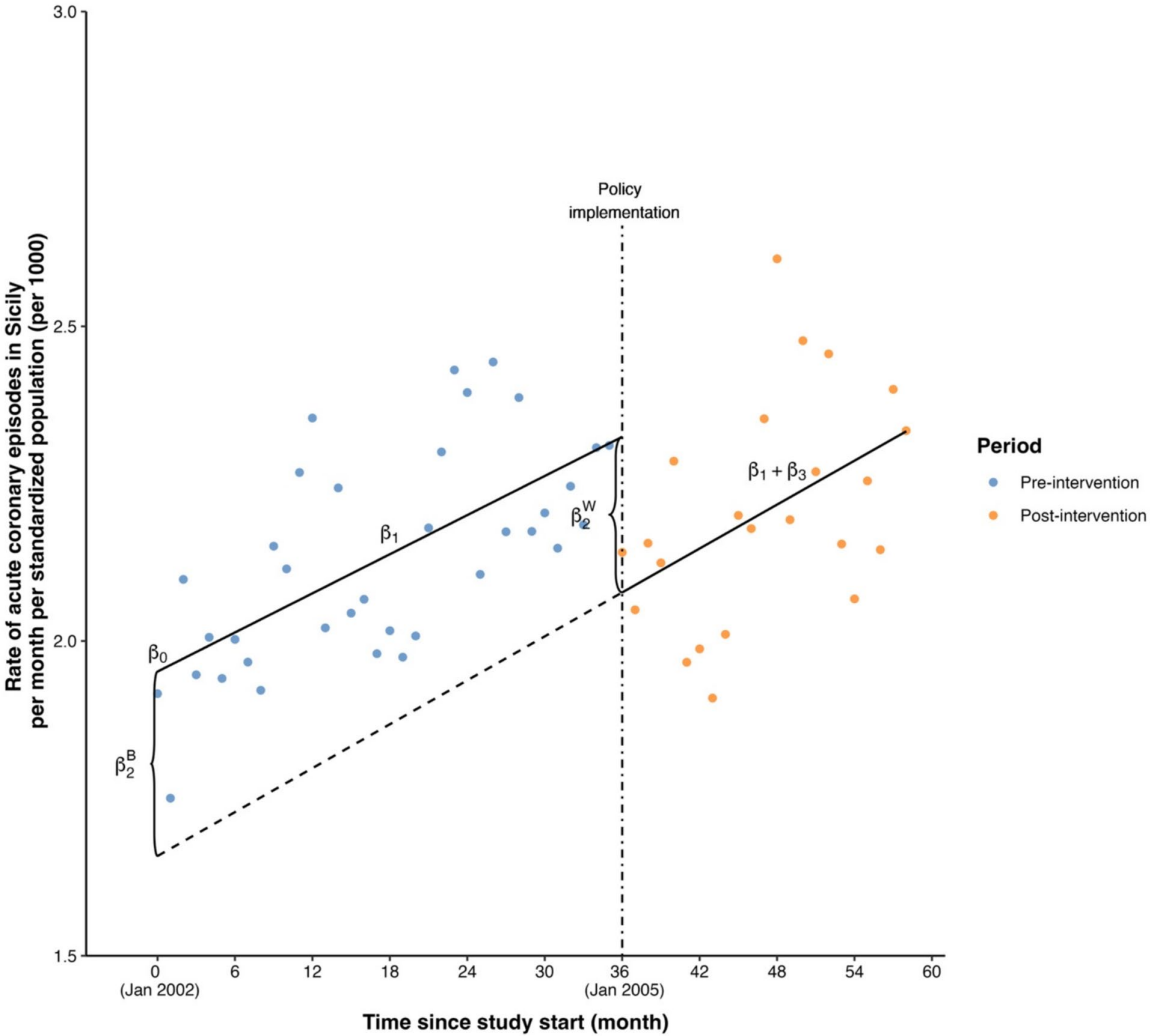
Estimated segmented regression line using the illustration dataset. The scatter points in blue and orange represented the data points in the pre- and post-intervention periods, respectively, while the black lines represented the pre- and post-intervention models. The vertical black dot-dashed line represented the time point of policy (i.e., intervention) implementation. The black dashed line represented the extension of the post-intervention model

Table 2 displays estimated coefficients and intervention effects and standard errors calculated as described in previous sections. Figure 1 displays the fitted model. The supplementary materials include implementation details with R code. ${\beta }_{0}$ is the intercept of the pre-intervention model and corresponds to the standardized rate of ACE per 1000 in January 2002, estimated as 1.95 (SE 0.05). ${\beta }_{1}$ is the slope of the pre-intervention model and indicates that the standardized rate of ACE per 1000 was increasing an estimated 0.01 units (SE 0.002) per month during this interval. At the time of intervention onset, it is estimated that the standardized rate of ACE per 1000 had dropped by 0.25 units (SE 0.08), corresponding to an immediate intervention effect; the decrease was statistically significant (*p* = 0.002). Thereafter, the standardized ACE rate per 1000 continued to increase at an estimated rate of 0.01per month (SE 0.004). The difference in slopes before and after intervention onset was not significantly different from zero, indicating no evidence of a gradual intervention effect.

**Table 2:** Estimated coefficients^a^ in segmented regression with standard errors (SE) and P-values

**Interpretations**	**Coefficients**		**Estimate (SE)**	***P*-value**
	**Bernal’s parametrization**	**Wagner’s parametrization**		
Baseline level	${\beta }_{0}$		1.95 (0.05)	< 0.0001
Pre-intervention trend	${\beta }_{1}$		0.01 (0.002)	< 0.0001
Difference in intercepts	${\beta }_{2}^{B}$	${\beta }_{2}^{W}-{\beta }_{3}\delta$	− 0.29 (0.22)	0.1840
Immediate effect (change in levels at intervention onset)	${\beta }_{2}^{B}+{\beta }_{3}\delta$	${\beta }_{2}^{W}$	− 0.25 (0.08)	0.0018
Gradual effect (change in slopes after intervention)	${\beta }_{3}$		0.001 (0.005)	0.8012
Post-intervention trend	${\beta }_{1}+{\beta }_{3}$		0.01 (0.004)	< 0.0001

^a^The estimated coefficients were derived from segmented regression, with the model equation representing standardized ACE rates per 1000 regressed on continuous time, a binary indicator denoting the presence or absence of intervention implementation, and their interaction term

The difference in estimates of ${\beta }_{2}$ between the two parametrizations of segmented regression is noteworthy. Figure 1 visually illustrates the difference between two estimated ${\beta }_{2}$ values. ${\widehat{\beta }}_{2}^{W}$ corresponds to the difference in the fitted outcome value at the time of intervention onset between the pre- and post-intervention models (immediate effect), represented as the vertical distance between the two regression lines at that time point. In contrast, ${\widehat{\beta }}_{2}^{B}$ is the difference in intercepts between the pre- and post-intervention models. In this dataset, the two quantities have similar values. This is because there is little difference in slopes between the pre- and post-intervention intervals. In data in which the two slopes are different, we would expect to see a greater difference between these two values.

## Discussion

In our investigation of the two common parametrizations of segmented regression for ITS, we verified that the coefficients for baseline outcome level, pre-intervention trend, and difference in slopes pre- and post-intervention onset are the same for both parametrizations. However, the interpretation of the coefficient for the binary intervention indicator differs between the two parametrizations. Under Wagner’s parametrization, this coefficient captures the difference in mean outcome between the pre- and post-intervention models at the time of intervention implementation, indicating the change-in-level or immediate effect. Under Bernal’s parametrization, this coefficient is not the immediate effect but rather captures the difference in the intercept between the pre- and post-intervention models. Unfortunately, this coefficient has sometimes been misinterpreted in the literature [20, 29–39].

When employing Bernal’s parametrization in segmented regression, it is important to recognize that the immediate effect should be calculated as a combination of two coefficients, as we have described. Conversely, when applying Wagner’s parametrization, the coefficient associated with the binary intervention indicator can be used as an estimate of the immediate effect and to get the difference in intercepts, one needs to use a combination of two coefficients. Thus, Bernal's parametrization is more convenient for computing the difference in intercepts, while Wagner's parametrization is more convenient for immediate effects. Users can choose between these parametrizations to tailor their estimates. Regardless of the chosen parametrization, both approaches yield the same pre- and post-intervention models. Both approaches are based on linear models, offering a flexible framework that allows for addressing potential confounders through methods such as covariate adjustment, stratification, subsetting, or other approaches. For example, propensity score-based ITS is discussed by Linden et al. [25].

ITS analysis is most straightforward to apply when a single well-defined intervention begins full implementation at a single well-defined timepoint. Our illustration, involving a smoking ban with a specific start date, meets these criteria. ITS methods have also been applied in situations in which there are multiple exposure periods; for example, Jeffery et al. (2024), or staggered adoption across multiple units; for example, Antonelli and Beck (2023) [40, 41]. Extensions of the standard ITS segmented regression model have been developed for these situations. Other extensions involve using a penetration variable which quantifies the extent to which the intervention has penetrated or been implemented across the relevant unit (Huitema et al. 2014) [42]. A penetration variable allows for a more nuanced, quasi-continuous estimate of the treatment effect.

Both of the parametrizations we have discussed have limitations. They both hypothesize an outcome change immediately after intervention implementation and a linear change over time both before and after the intervention implementation. However, these assumptions might not accurately represent the dynamics of the study; for example, intervention effects can exhibit lagged impacts. In such cases, one can consider alternative parametrizations that incorporate delayed effects or include a transition period between pre-intervention and post-intervention periods [6, 16]. Numerous technical issues related to segmented regression, such as autocorrelation, seasonality, and heterogeneity, have been addressed in existing literature [1, 2, 4, 5, 15, 16, 22]. By applying segmented regression and selecting appropriate parametrizations, users can employ tailored tools to mitigate technical issues based on the specifics of their data. The last issue to consider in ITS studies relates to the setup for causal estimation. Since ITS designs—whether with or without control groups—are often utilized when randomized trials are infeasible, they are well-suited for large-scale observational databases such as electronic health records. In this context, causal inference methods like target trial emulation (TTE) can offer a more robust framework by approximating the conditions of randomization within an observational study design [43, 44]. However, health services, policy research, and public health studies frequently rely on population-level outcomes over time (e.g., cancer screening rates, health insurance enrollment rates), where individual-level data may be unavailable or not central to the research question [29–35, 37, 39, 45]. When individual-level data are accessible, integrating ITS with TTE can strengthen causal inference. This combined approach allows ITS to capture population-level intervention or policy effects over time, while TTE provides individual-level effect estimates, adjusting for a broader range of potential confounders [46]. While ITS designs offer valuable insights for evaluating interventions and policies in real-world settings, careful consideration of causal estimation strategies is essential to enhance the validity of findings. The integration of complementary methods, such as TTE when feasible, can provide a more comprehensive understanding of intervention effects across both population and individual levels, ultimately advancing the rigor and impact of health services and policy research.

## Conclusion

In conclusion, two common segmented regression parametrizations in ITS analysis represent the same model, yielding identical pre- and post-intervention models but distinct coefficient interpretations. Immediate intervention effect calculations differ between parametrizations, while gradual intervention effect calculations remain consistent. Both parametrizations for segmented regression can be employed as analytical approaches for ITS design, provided the specific nuances and interpretations of the coefficients are understood and explained.

## Supplementary Information

Supplementary Material 1.

## Data Availability

No datasets were generated or analysed during the current study.

## Abbreviations

ITS: Interrupted time series
ACE: Acute coronary episodes
TTE: Target trial emulation
