# Supplementary material for: Interpretation of coefficients in segmented regression for interrupted time series analyses
Source: BMC Med Res Methodol. 2025 Apr 16;25:98. doi: 10.1186/s12874-025-02556-8 (PMC12001611; doi:10.1186/s12874-025-02556-8)
Supplement: Supplementary file 1 — Supplementary Material 1. [file 12874_2025_2556_MOESM1_ESM.pdf]

Supplementary

1. Dataset

```
# Read data
Dt <- read.csv('sicily.csv')
# Compute the standardized rates
Dt$rate <- Dt$aces / Dt$stdpop * 1000
Dt$time <- Dt$time - 1
# View data
head(Dt)
```

```
##   year month aces time smokban      pop   stdpop     rate
## 1 2002     1  728    0       0 364277.4 379875.3 1.916418
## 2 2002     2  659    1       0 364277.4 376495.5 1.750353
## 3 2002     3  791    2       0 364277.4 377040.8 2.097916
## 4 2002     4  734    3       0 364277.4 377116.4 1.946349
## 5 2002     5  757    4       0 364277.4 377383.4 2.005918
## 6 2002     6  726    5       0 364277.4 374113.1 1.940590
```

2. Model

(1). Estimation setup

```
# Number of observations in pre- and post-intervention periods
m <- table(Dt$smokban)[1]
n <- table(Dt$smokban)[2]
N <- m + n

# Design matrix
Time <- Dt$time
A <- sum(Time[1:m])
B <- sum(Time[1:m]^2)
C <- sum(Time[(m + 1):N])
D <- sum(Time[(m + 1):N]^2)

# Outcome
Y <- Dt$rate
Delta <- sum(Y[1:m])
Omega <- sum(Time[1:m] * Y[1:m])
Lambda <- sum(Y[(m + 1):N])
Eta <- sum(Time[(m + 1):N] * Y[(m + 1):N])
```

```
# Timepoint of intervention
delta <- m
```

(2). Estimated coefficients and their standard errors from analytical forms

```
# Design matrix
X <- model.matrix(~ time * smokban, data = Dt)

# Beta 0, 1, 3
Beta0 <- (Omega * A - Delta * B)/(A^2 - m * B)
Beta1 <- (Delta * A - m * Omega)/(A^2 - m * B)
Beta3 <- (Lambda * C - n * Eta)/(C^2 - n * D) - Beta1

# Beta 2
## Bernal's parametrization
Beta2_a <- (Eta * C - Lambda * D)/(C^2 - n * D) - Beta0

## Wagner's parametrization
Beta2_c <- Beta2_a + delta * Beta3

# Estimated residuals
Coef <- rbind(Beta0, Beta1, Beta2_a, Beta3)
Yhat <- X %*% Coef
sigma <- 1/(N - 3 - 1) * sum((Y - c(Yhat))^2)

# Estimated standard errors
Beta0_se <- sqrt(- B/(A^2 - m * B) * sigma)
Beta1_se <- sqrt(- m/(A^2 - m * B) * sigma)
Beta3_se <- sqrt((- m/(A^2 - m * B) - n/(C^2 - n * D)) * sigma)
Beta2_a_se <- sqrt((- B/(A^2 - m * B) - D/(C^2 - n * D)) * sigma)
Beta2_c_se <- sqrt(((2 * delta * C - D - delta^2 * n)/(C^2 - n * D) +
                      (2 * delta * A - B - delta^2 * m)/(A^2 - m * B)) * sigma)

Estimated_Coef <- data.frame('Estimate' = c(Beta0, Beta1, Beta3, Beta2_a, Beta2_c),
                             'Std.Error' = c(Beta0_se, Beta1_se, Beta3_se, Beta2_a_se, Beta2_c_se))
rownames(Estimated_Coef) <- c('Intercept', 'time', 'smokban * time',
                              'smokban_Bernal', 'smokban_Wagner')
Estimated_Coef
```

```
##                    Estimate   Std.Error
## Intercept       1.951445891 0.046523391
## time            0.010358486 0.002286040
## smokban * time  0.001272204 0.005028733
## smokban_Bernal -0.292970791 0.217633900
## smokban_Wagner -0.247171431 0.075251715
```

(3). Estimated coefficients from lm() function

```r
# Design matrix without intercept column
## Bernal's parametrization
Xa <- model.matrix(~ time * smokban - 1, data = Dt)
m1 <- lm(Y ~ Xa)
s1 <- summary(m1)$coefficients[, 1:2]

## Wagner's parametrization
Xc <- Xa
Xc[which(Xc[, 2] == 1), 3] <- Xc[which(Xc[, 2] == 1), 3] - delta
m3 <- lm(Y ~ Xc)
s3 <- summary(m3)$coefficients[, 1:2]

Estimated_Coef_lm <- as.data.frame(rbind(s1[c(1, 2, 4), ], s1[3, ], s3[3, ]))
rownames(Estimated_Coef_lm) <- c('Intercept', 'time', 'smokban * time',
                                 'smokban_Bernal', 'smokban_Wagner')
Estimated_Coef_lm
```

```
##                    Estimate  Std. Error
## Intercept       1.951445891 0.046523391
## time            0.010358486 0.002286040
## smokban * time  0.001272204 0.005028733
## smokban_Bernal -0.292970791 0.217633900
## smokban_Wagner -0.247171431 0.075251715
```
